# *In vitro* models for investigating itch

**DOI:** 10.3389/fnmol.2022.984126

**Published:** 2022-10-26

**Authors:** Hendrik Mießner, Judith Seidel, Ewan St. John Smith

**Affiliations:** ^1^Department of Pharmacology, University of Cambridge, Cambridge, United Kingdom; ^2^Dermatological Skin Care, Beiersdorf AG, Hamburg, Germany

**Keywords:** itch, *in vitro*, preclinical, surrogate model, pruritus, induced pluripotent stem cell, atopic dermatitis, sensory neuron

## Abstract

Itch (pruritus) is a sensation that drives a desire to scratch, a behavior observed in many animals. Although generally short-lasting and not causing harm, there are several pathological conditions where chronic itch is a hallmark symptom and in which prolonged scratching can induce damage. Finding medications to counteract the sensation of chronic itch has proven difficult due to the molecular complexity that involves a multitude of triggers, receptors and signaling pathways between skin, immune and nerve cells. While much has been learned about pruritus from *in vivo* animal models, they have limitations that corroborate the necessity for a transition to more human disease-like models. Also, reducing animal use should be encouraged in research. However, conducting human *in vivo* experiments can also be ethically challenging. Thus, there is a clear need for surrogate models to be used in pre-clinical investigation of the mechanisms of itch. Most *in vitro* models used for itch research focus on the use of known pruritogens. For this, sensory neurons and different types of skin and/or immune cells are stimulated in 2D or 3D co-culture, and factors such as neurotransmitter or cytokine release can be measured. There are however limitations of such simplistic *in vitro* models. For example, not all naturally occurring cell types are present and there is also no connection to the itch-sensing organ, the central nervous system (CNS). Nevertheless, *in vitro* models offer a chance to investigate otherwise inaccessible specific cell–cell interactions and molecular pathways. In recent years, stem cell-based approaches and human primary cells have emerged as viable alternatives to standard cell lines or animal tissue. As *in vitro* models have increased in their complexity, further opportunities for more elaborated means of investigating itch have been developed. In this review, we introduce the latest concepts of itch and discuss the advantages and limitations of current *in vitro* models, which provide valuable contributions to pruritus research and might help to meet the unmet clinical need for more refined anti-pruritic substances.

## Introduction

### Medical relevance of itch

Itch is dismissed by many as an unpleasant yet well-treatable result of insect bites, but is considered a major morbidity in numerous highly prevalent chronic inflammatory skin diseases, such as atopic dermatitis (AD) or itchy psoriasis (Silverberg et al., 2018; Elewski et al., 2019). Other medical causes of itch include side effects of prescription drugs (e.g., chloroquine, opioids), neurological (e.g., brain lesions, multiple sclerosis), autoimmune (e.g., lupus erythematosus, systemic sclerosis), and liver diseases (cholangitis, cholestasis; Weisshaar, 2016; Zeidler et al., 2019; Pereira et al., 2021). Unlike the sensation after insect bites, chronic itch, or pruritus, is more complex, not yet fully understood, and treatment options are limited. It affects up to 20% of the population at least once in their lifetime, severely impacting quality of life (Weisshaar, 2016, 2021).

Scratching-induced pain suppresses the feeling of itch and results in instant relief, a deceptive pleasure causing damage that, in AD for example, disrupts the skin, exacerbates inflammatory symptoms, and leads to bleeding and scarring (Furue et al., 2017; Yosipovitch et al., 2019). Chronic itch patients also commonly experience psychological problems owing to itch-induced sleep deprivation and body image insecurity (Darlenski et al., 2014; Mann et al., 2020).

On average, chronic pruritus patients lose 5.5 quality-adjusted life years and take on lifetime treatment costs of 274,921 USD (Whang et al., 2021). Needless to say, chronic itch poses both a heavy health and economic burden on affected groups underlining the need for better drug development and patient care. More research is needed to resolve this situation and suitable means for experimental investigation are required to rapidly advance progress. We believe that *in vitro* models of itch can majorly contribute to a better mechanistic understanding of diseases associated with this society-wide problem.

### Cellular and molecular basis of itch

Itch likely evolved as a helpful defense mechanism against pathogens and vermin (e.g., bugs, mites), triggered by diverse stimuli (Wimalasena et al., 2021). These signals get picked up by different cells in the body, eventually being transmitted by sensory neurons to the brain, where the urge to scratch arises.

**Figure 1:**
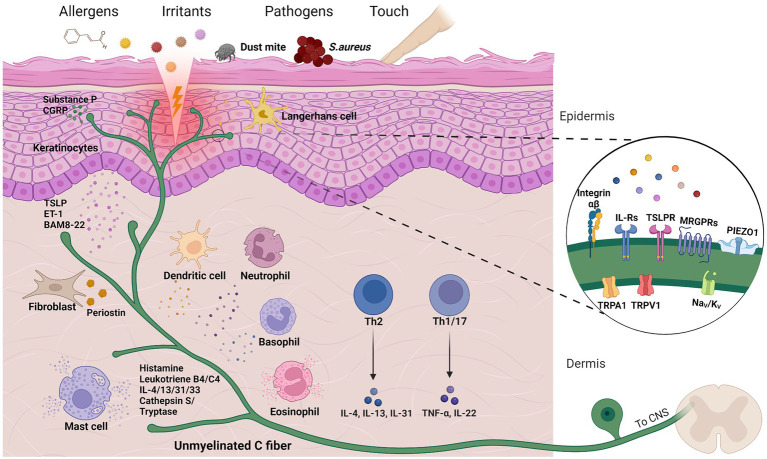
Cells and molecules in cutaneous itch transmission. Chemical or mechanical triggers can activate sensory neurons to produce the sensation of itch, a process characterized by intercellular crosstalk and both skin and immune cell activation. A selection of pruritogen receptors is depicted in close-up. Created with Biorender.com.

To assess the recent contributions to the field of itch, it is essential to first introduce how itch develops on a cellular level (Figure 1). It begins with environmental stimuli, pathogens or any other itch-causing substances, also called pruritogens, which enter the epidermis and encounter keratinocytes, dendritic cells or directly activate free nerve endings. Upon contact, skin cells and resident immune cells release cytokines that target neighboring sensory nerves (so-called pruriceptors), which express pruritogen receptors that upon activation initiate diverse signaling cascades (Dong and Dong, 2018; Kahremany et al., 2020; Wang and Kim, 2020). This ultimately causes depolarization, action potentials and neurotransmitter release (e.g., glutamate, substance P or B-type natriuretic peptide) for signal transmission in the spinal cord. In the dorsal horn of the spinal cord, gastrin-releasing peptide (GRP)-positive neurons act as a relay hub for itch propagation; the importance of these neurons is demonstrated by a failure of pruritogen-induced itching behavior in mice lacking GRP-positive neurons (Sun and Chen, 2007; Sun et al., 2009). Finally, projection neurons transmit the itch signal along the spinothalamic tract to the brainstem where the signal unfolds to further brain regions and the urge to scratch develops (Dong and Dong, 2018; Chen and Sun, 2020). For detailed information on itch circuits and processing in the central nervous system and brain in particular, the following recent reviews are recommended (Chen and Sun, 2020; Najafi et al., 2021; Mu and Sun, 2022).

However, the above brief overview of itch transmission greatly simplifies the complexity of itch by leaving out a variety of factors and cell types, which will be discussed in more detail below. Itch is an inherent component of many of the medical conditions mentioned previously and/or can be triggered by diverse endogenous or environmental stimuli, such as irritants, allergens or emotional stress (Murota and Katayama, 2017; Golpanian et al., 2020). Adding to this, the ability of pruritogens to interact with pruriceptors and other cell types can be facilitated by a damaged skin barrier. The transmission itself is also multi-faceted, dependent on various signaling cascades and neuronal subsets with differential receptor expression (Figure 1). Therefore, it is crucial to understand what exactly triggers itch, the cell types involved, and critical mediators and receptors for accurate treatment options. To highlight the multitude of disease-associated factors, Table 1 summarizes essential interactions between pruritogen receptors and endogenous mediators involved in several pruritic conditions. Beyond the scope of this review, emerging treatment options have been discussed in other recent reviews (Misery et al., 2021; Kim, 2022).

**Table 1:** Key sensory neuron pruritogen receptors and their endogenous ligand sources.

Pruritogen receptor	Ligand	Ligand-producing cells	Diseases (selection)
IL-31RA	IL-31	Th2 (Dillon et al., 2004), mast cells (Niyonsaba et al., 2010)	AD (Silverberg et al., 2021), ACD (Takamori et al., 2018), psoriasis (Nattkemper et al., 2018), PN (Hashimoto et al., 2021b)
IL-4R / IL-13RA1	IL-4 / IL-13	Th2 (Kortekaas Krohn et al., 2022), mast cells (Mcleod et al., 2015)	AD (Simpson et al., 2016), ACD (Neis et al., 2006), PN (Holm et al., 2020)
OSMR	OSM	T cells, neutrophils (Tseng and Hoon, 2021), monocytes/macrophages (Mommert et al., 2020), dendritic cells (Suda et al., 2002)	AD (Mikhak et al., 2019), psoriasis, cutaneous T cell lymphoma (Tseng and Hoon, 2021), PN (Hashimoto et al., 2021b)
MOR	β-endorphin	Keratinocytes (Bigliardi-Qi et al., 2004), T cells (Labuz et al., 2010), other immune cells (Mousa et al., 2004)	AD (Ádám et al., 2022), psoriasis (Taneda et al., 2011), PN (Weisshaar et al., 2022), uremic pruritus (Fishbane et al., 2020)
H1R/H4R	Histamine	Mast cells (Fawcett, 1954), T(h2) cells (Gutzmer et al., 2009), other immune cells but mast cells are the greatest source (Togias, 2003)	Acute itch (e.g., insect bites; Fostini et al., 2019), AD (Albrecht and Dittrich, 2015)
TSLPR	TSLP	Keratinocytes (Wilson et al., 2013b), fibroblasts, dendritic cells (reviewed in Takai, 2012)	AD (Berna et al., 2021), PN (Zhong et al., 2019)
PAR2	Tryptase, Cathepsin S	Mast cells (Siiskonen and Harvima, 2019), basophils (Jogie-Brahim et al., 2004); Crucial PAR2 expression: Keratinocytes (Zhao et al., 2020)	AD (Nattkemper et al., 2018)
MRGPRX1	Enkephalins (BAM 8-22)	Skin source not clear (only for the precursor proenkephalin A); fibroblasts, keratinocytes (Slominski et al., 2011)	ACD (Li et al., 2022), acute tick itch (Li et al., 2021), cholestatic pruritus (Sanjel et al., 2019), psoriasis (Slominski et al., 2011)
Integrin α_V_β_3_	Periostin	Fibroblasts (Masuoka et al., 2012), keratinocytes (Rosselli-Murai et al., 2013)	AD (Kou et al., 2014), PN (Hashimoto et al., 2021c)
5-HT7/5-HT3	Serotonin (5-HT)	Fibroblasts, keratinocytes (Slominski et al., 2002), but mostly chromaffin cells (Banskota et al., 2019)	AD (Morita et al., 2015), cholestatic pruritus (Schwörer et al., 1995)

Indirect receptors and channels (e.g., transient receptor potential family, Janus kinases) involved in itch transmission are not included. ACD, allergic contact dermatitis; AD, atopic dermatitis; PN, prurigo nodularis; 5-HT, 5-hydroxytryptamine; BAM, bovine adrenal medulla; MOR, μ opioid receptor; MRGPRX1, Mas-related G protein-coupled receptor X1; OSM(R), oncostatin M (receptor); PAR2, protease-activated receptor 2; TSLP(R), Thymic stromal lymphopoietin (receptor).

## Cell types and receptors involved in itch

### Histamine-dependent itch

The simplest example for an urge to scratch follows a mosquito bite that causes a well-researched, histamine-dependent itch, also referred to as acute itch. Histamine released from mast cells activates histamine-1-(H1R) and histamine-4-receptors (H4R) on unmyelinated, histamine-responsive C-fibers (Yosipovitch et al., 2018). That is why H1R blockers (typically called “antihistamines”) work well to specifically treat this type of itch. Although histamine-evoked itch has been demonstrated in many species, naked mole-rats (*Heterocephalus glaber*) are behaviorally non-responsive to histamine despite their peripheral sensory neurons being activated by histamine (Smith et al., 2010). The demonstration that intrathecal Substance P administration could rescue histamine-evoked scratching highlights that although peripheral pruriceptors are important, the anatomical pathways and chemical mediators regulating itch transmission are complex and likely species-specific. For mice, histamine injection into the cheek (not neck) with visible hind limb scratching seemed to distinguish between pain and itch responses (Shimada and Lamotte, 2008; Lamotte et al., 2011). This site-directed differentiation is essential for interpreting mouse behavioral data and extrapolating to the human system.

### Histamine-independent itch – The contribution of skin and immune cells

Histamine-independent itch often occurs in severe, relapsing pathological states, therefore often being described as chronic itch. In chronic disease conditions, the activation of pruriceptors is more intricate. This is because multiple processes are at work, from immune cell cytokine release to nerve interaction with skin cells, resulting in both peripheral itch-transmission and possibly even long-term sensitization to pruritogens or normally innocuous stimuli (Jin and Wang, 2019). In recent years, the link between immune and nervous systems has gained increasing attention and current research suggests that many cell types are involved in the generation of pruritus signals (Oetjen and Kim, 2018; Yang and Kim, 2019; Tauber et al., 2021). Neuroimmune interactions are even more important in inflamed skin, where resident and immigrating (immune) cells act in concert, creating a microenvironment of chemical mediators and pruritogens that bathe sensory neurons.

### Structural skin cells

Considering that itch arises from the skin, it is no surprise that fibroblasts and keratinocytes are important not only for skin homeostasis, but also for itch. Epidermal keratinocytes directly interact with intraepidermal sensory nerves via synapse-like structures and cytoplasmic tunnels (Talagas et al., 2020a,b). Through ATP release, keratinocytes are thought to contribute to the perception of touch, heat and cold, via activation of P2X receptors expressed by sensory neurons (Moehring et al., 2018; Sadler et al., 2020; Shindo et al., 2021). Keratinocytes also transduce itch via secretion of thymic stromal lymphopoietin (TSLP), a typical AD and Th2-associated cytokine that is expressed upon exposure to TNF-α and various allergens (Takai, 2012; Wilson et al., 2013b; Mizuno et al., 2015). Stimulation of keratinocyte toll-like receptors (TLR) 2-6 by pathogens (e.g., *Staphylococcus aureus*) or scratching (in case of TLR3) and house dust mite (HDM)-induced activation of protease-activated receptor 2 (PAR2) also significantly contribute to pruritus/AD via TSLP secretion (Takai et al., 2014; Smith et al., 2019; Szöllősi et al., 2019; Buhl et al., 2020). Located underneath the epidermis, fibroblasts are an integral cellular part of the dermis. Fibroblasts actively shape the extracellular matrix (ECM), the environment through which sensory neurons navigate to reach their destined structure, such as hair shafts (Myers et al., 2011; Long and Huttner, 2019). ECM guidance proteins include laminins, for basement membrane interaction, and the adhesion molecule periostin. Dermal periostin is upregulated in patients with both AD and prurigo nodularis (PN, a chronic, inflammatory skin condition characterized by itchy nodules), and also induces allergic itch in mice, dogs and non-human primates via integrin α_V_β_3_ (Kou et al., 2014; Sung et al., 2017; Mishra et al., 2020; Hashimoto et al., 2021c). Through perturbed periostin homeostasis, fibroblasts could therefore play a key role in pruritus pathology (Hashimoto et al., 2021a; Ono et al., 2021).

### Mast cells and other granulocytes

Mast cells are predominantly known for their role in histaminergic itch, but also secrete other pruritogens such as the lipid mediators leukotriene B4 and C4, itch-associated cytokines IL-4, 13, 31, 33 and proteases (tryptase, cathepsin S) that activate PAR2 (Toyama et al., 2021).

Similar to mast cells, basophils (Hashimoto et al., 2019), eosinophils (Radonjic-Hoesli et al., 2021), neutrophils (Hashimoto et al., 2018), dendritic cells and macrophages all share the ability to release pruritogenic substances and likely contribute to itch (Toyama et al., 2021), especially in immune cell-enriched inflamed skin (Nguyen and Soulika, 2019).

## T helper cells

### Atopic dermatitis

Th2 and Th1/17 cells take on a special role as key drivers for inflammation and itch in AD and psoriasis, respectively. Th2 cells prominently secrete IL-4, -13 and -31, the typical AD cytokines involved in itch induction in mice and humans (Dillon et al., 2004; Campion et al., 2019). Research revealed that these cytokines can directly activate their respective receptors on human and mouse primary sensory neurons (Cevikbas et al., 2014; Oetjen et al., 2017). Their central role in pruritus was further underlined by the newly developed anti-IL-4 receptor-α (IL4Rα, also subunit of IL13R) antibody dupilumab for moderate to severe cases of AD, which has proven greatly effective (Simpson et al., 2016; Le Floc’H et al., 2020; Simpson et al., 2020). However, lifetime costs of dupilumab treatment are estimated at 0.5 million USD/person, thus too high to become the standard treatment for AD patients (Zimmermann et al., 2018).

The Th2 cytokine IL-31 is especially interesting for pruritus research, as it seemingly causes itch that is uncoupled from inflammation (Takamori et al., 2018). In humans, skin-prick testing of IL-31, unlike histamine, causes late-onset itch after more than 60 min (Hawro et al., 2014b). Clinical trials with the anti-IL-31 receptor alpha (IL31RA) antibody nemolizumab appear highly effective in reducing pruritus and AD symptoms (Kabashima et al., 2020; Silverberg et al., 2020, 2021; Kabashima and Irie, 2021). The receptor for IL-31 is a heterodimer composed of IL31RA and the oncostatin M receptor beta (OSMR). Therefore, unsurprisingly, oncostatin M also plays a role in pruritus and inflammation (Pohin et al., 2016; Tseng and Hoon, 2021). In the first clinical trial, KPL-716/vixarelimab, a monoclonal antibody against OSMR, reduced pruritus in AD patients (Mikhak et al., 2019). One further mechanism contributing to itch in AD may be an imbalance in opioid receptor expression (Ádám et al., 2022). This is because binding of β-endorphin to the μ-opioid receptor (MOR) causes itch, while activation of κ-opioid receptor (KOR) suppresses it; the following review provides a detailed discussion of opioid receptor signaling in relation to itch (Nguyen E. et al., 2021).

### Psoriasis

Even though non-itchy psoriasis exists, pruritus is still a burden for most patients, but knowledge is somewhat limited compared to our understanding of AD (Elewski et al., 2019). Th1/17 cells are the main source of psoriasis cytokines IL-17, 22, TNF-α and interferon-γ. Resident T cells are crucial for the induction of psoriasiform inflammation and skin lesions, even when induced *ex vivo* (Gallais Serezal et al., 2018, 2019). However, in contrast to AD, these cytokines do not generally activate sensory neurons directly (Komiya et al., 2020). It has however been shown that TNF-α potentiates other forms of itch and the TNF-α sequestering agent etanercept reduced dry skin itch, thus underlining its use as a treatment option in psoriasis (Miao et al., 2018). As mentioned for AD, there is evidence for altered opioid receptor signaling in various types of chronic itch, including psoriasis, for example, expression of KOR and its ligand dynorphin A are reduced in psoriatic lesions (Taneda et al., 2011).

## Itch signaling receptors

### Transient receptor potential channels

Activation of itch-sensing pruritogen receptors is often not directly responsible or sufficient for itch transmission. Instead, pruritogen receptors often rely on secondary channel openings for Ca^2+^-influx, action potential generation and signal propagation. Of particular interest with regard to both itch and pain are certain members of the transient receptor potential (TRP) ion channel family (Sun and Dong, 2016; Feng et al., 2017). Specifically for itch, those with the most well characterized role are TRP vanilloid 1, 3 (TRPV1, TRPV3) and ankyrin 1 (TRPA1; Wilson et al., 2013a; Kittaka and Tominaga, 2017). Known pruritogens such as histamine, IL-31, -13 and TSLP all signal through TRPV1 or TRPA1, which are expressed in subsets of sensory neurons (Oh et al., 2013; Wilson et al., 2013b; Cevikbas et al., 2014; Sun and Dong, 2016; Wilzopolski et al., 2021). By contrast, TRPV3 activation happens in keratinocytes and plays a pivotal role for itch in skin conditions like AD, psoriasis and post-scar itch (Park et al., 2017; Seo et al., 2020; Larkin et al., 2021). Moreover, TRPV3 plays a dominant role in HDM-mediated itch via a PAR2/TRPV3/TSLP pathway in keratinocytes (De Boer et al., 2014; Zhao et al., 2020). Other TRP channels, such as TRPC4 and TRPV4, have also been reported to play a role in pruritus (Lee S. H. et al., 2020; Zhang et al., 2021). It is further speculated that TRP channel sensitization by Th2 cytokines could play a crucial role in AD severity and manifestation of the inflammatory-itch-axis (Meng et al., 2021). However, although long known to be important in pruritus, clinical trials for itch disorders with TRP antagonists have been scarce. One limitation of targeting many TRP channels is their broad biological functions, including temperature-sensing and pain transmission making them a difficult target when considering potential side effects (Xie and Hu, 2018; Koivisto et al., 2022). Nevertheless, a novel TRPA1 inhibitor (GDC-0334) improved pain and itch in a Phase I study designed for asthma treatment (Balestrini et al., 2021). In addition, the TRPV1 antagonist asivatrep recently passed a Phase 3 clinical study with AD patients (Park et al., 2022), thus demonstrating the significant therapeutic potential of targeting TRP channels in pruritus research.

### Other forms and mediators of itch

As well as pathways involving TRP channel activation, common itch-associated cytokine receptors often signal through the Janus kinase/signal transducer and activator of transcription (JAK/STAT) pathway in neurons (Leonard, 2001; Oetjen et al., 2017). Examples for this are IL-4, -13, -31 and TSLP. Selective JAK1/2 pathway inhibition has resulted in significant improvements for AD patients (Kim et al., 2020; Tsai et al., 2021) and even in dogs, the JAK1/2 inhibitor oclacitinib is used for treating AD (Cosgrove et al., 2013).

Activation of the G protein-coupled receptor family named mas-related G-protein coupled receptors (MRGPRs) also causes histamine-independent itch (Meixiong et al., 2019). For example, the food supplement β-alanine activates MRGPRD and causes itch in both mice and humans when injected intradermally (Liu et al., 2012). Adding to this, the antimalarial drug chloroquine induces severe pruritus in some individuals via MRGPRX1 activation (Liu et al., 2009; Liu and Dong, 2015). MRGPRX1 could play a role in cholestatic itch as well, both genes for MRGPRC11 (rodent analog of MRGPRX1) and proenkephalin, the precursor of the endogenous MRGPRX1-ligand bovine adrenal medulla peptide 8-22 (BAM8-22), are upregulated in a cholestasis mouse model (Sanjel et al., 2019). Cholestatic pruritus however is a poorly understood multifactorial itch condition, speculated to involve TRP channels (Langedijk et al., 2021). For example, lysophosphatidylcholine (LPC) activates TRPV4, leading to the release of microRNA-146a that causes itch by targeting sensory neurons, matching the elevated concentrations of both LPC and microRNA-146a in cholestatic itch patients (Chen et al., 2021).

Serotonergic itch mediated via 5-hydroxytryptamine receptors (HTR) is also linked to cholestatic pruritus (Tian et al., 2016), given that 5-HT_3_ receptor antagonists provide itch relief for selected patients (Schwörer et al., 1995). Serotonergic itch might also be associated with AD, but thought to involve 5-HT_7_ receptors acting in concert with TRPA1 (Morita et al., 2015). Notably, MRGPRs are also known to act through TRPA1 (Wilson et al., 2011).

While the mechanisms above describe chemically-induced itch, recent findings have shed new light on mechanical itch, which possibly evolved as a warning of crawling parasites. In mice, loss of function in PIEZO1, a mechanosensitive ion channel, prevented mechanical itch and sensory neuron knockout of PIEZO1 also reduced spontaneous scratching bouts in hypersensitive AD mice models, further demonstrating the complexity of chronic itch (Hill et al., 2022). Of note, if mechanical itch mechanisms also play a role in chronic itch, the sub-epidermal grid of mechanosensitive, non-myelinating Schwann cells could be involved. These cells have been found to transmit pain sensation via TRPA1 and could therefore theoretically modulate itch sensation as well (De Logu et al., 2017; Abdo et al., 2019). However, the roles of Schwann cells and PIEZO1 in itch require further investigation in humans.

## State of the art in itch research

Lab-based itch research has become a valuable tool to support clinical findings and pave the way for drug development. More elaborate means of cellular and molecular analyses alongside new itch models have been created with the goal to gain mechanistic insight into the cellular and molecular processes that underlie the complex pathology of itch.

### Identification of exclusive pruriceptors

Potentially of most importance in recent years was the identification of itch-specific sensory neurons that responded to multiple pruritogens and, when inhibited, did not influence nociception. As mentioned before, MRGPRs were identified as being involved in pruritus over a decade ago (Liu et al., 2009). However, MRGPRA3-positive sensory afferents were more recently identified as being crucial for itch sensation, such that their ablation reduced itch in a pruritus mouse model without noticeable impact on nocifensive behavior (Han et al., 2013; Qu et al., 2014). These exclusive pruriceptors were found to display intrinsic multimodality, a key concept in itch research. What this means is that slow metabotropic (Gq) activation of these neurons induced itch, whereas activation of ligand gated ion channels expressed by the same neurons, such as the ATP-gated P2X3 receptor, resulted in the sensation of pain (Sharif et al., 2020; Xing et al., 2020).

### Animal models of chronic itch have their limitations

Despite the exciting progress in itch research, it should be kept in mind however, that many insights presented throughout the previous sections are based on animal research and might not be entirely applicable to humans. A prime example being that MRGPRA3 does not exist in humans. The closest analog is MRGPRX1, the homolog for rodent MRGPRC11. However, C11 and X1 share BAM8-22 as agonist, but only X1 and A3 are activated by chloroquine (Tseng et al., 2019). In fact, the general cell composition/variety of human vs. mouse DRG differs quite significantly. For example, many human sensory neurons are both CGRP and P2X3R positive, markers normally used to either define peptidergic or non-peptidergic neurons, respectively, in rodents (Shiers et al., 2020). A special focus on TRP channels showed only 79% sequence homology between rodent and human TRPA1 (Lindsay and Timperley, 2020). This is adding to a proteome analysis that revealed only 80% general overlap between rat and human DRG neurons (Schwaid et al., 2018).

Bulk and single-cell transcriptomic data have also highlighted differences between rodent and human DRG gene expression patterns (Ray et al., 2018; Nguyen M. Q. et al., 2021). Based on the first unbiased classification of mouse DRG neurons (Usoskin et al., 2015), pruriceptors were mainly described as non-peptidergic subgroups 1-3 (NP1-3). The NP3 neurons were positive for the key pruritogen receptors IL-31R and OSMR, as well as their downstream effector JAK1 (Oetjen et al., 2017). In contrast to mouse, not one but two IL-31R/OSMR^+^ types of neurons were identified in human DRG, JAK1 being expressed in both subpopulations. Human pruriceptors also appear to exhibit greater polymodality, expressing genes involved in mechanosensitivity, such as *PIEZO2* in *IL31RA*-positive cells, not found in their rodent counterparts (Nguyen M. Q. et al., 2021; Tavares-Ferreira et al., 2022). This could indicate a connection to what is described as mechanical itch, an oversensitization reaction also found in chronic itch conditions (Lee H. et al., 2022; see also previous section “Other forms and mediators of itch”). Additionally, immunohistochemical comparison of DRG with a focus on nociceptors, thought to act as pruriceptors as well, emphasized caution for translating mouse experimental data to humans (Rostock et al., 2018). It is thus slowly becoming clear that for all the clear benefits of modeling itch in rodents that there are significant limitations.

If not through direct pruritogen stimulation in healthy animals, research has often been conducted with rodent models exhibiting some sort of inflammatory and/or dry skin condition, often referred to as a chronic itch or AD model. For example, MC903, a vitamin D3 analog (Li et al., 2006) or an acetone/ether/water (AEW) application is commonly used (Miyamoto et al., 2002). Challenging rodents with allergens over time can also induce contact dermatitis (Lamotte, 2016). For further information, more detailed reviews on rodent itch models are available (Lamotte et al., 2011; Wheeler et al., 2020; Donglang et al., 2021).

However, the morphology of less thick, hairy rodent skin without downward epidermal projections (rete ridges) is still fundamentally different to human skin (Wong et al., 2011). Structurally, human epidermis consists of 3-times more keratinocyte layers than mouse skin. Moreover, the cell type composition can differ (human dermal α/β T cells vs. mouse epidermal γ/δ T cells), and the top 100 skin-associated genes are only 30% similar between mouse and human (Gerber et al., 2014). The chemokine CCL27 for example, observed to be especially upregulated in AD and psoriasis (Kakinuma et al., 2003), was only found in the human top 100 list and normally recruits skin-homing T cells. Whereas filaggrin 1 and 2 (*FLG1/2*) seem to be conserved between human and mouse, other genes encoding for proteins with barrier function and pathogen resistance, such as dermcidin (*DCD*), secretoglobin 2A2/1D2 (*SCGB2A2 and SCGB1D2*) and IL-37 (*IL37*), were exclusively found in human tissue without rodent homolog (Gerber et al., 2014). In fact, a general transcriptomic comparison between mouse and human also demonstrated substantial differences that likely limit the inter-species translatability of many biological findings in itch research (Breschi et al., 2017).

### Need for human models

Species differences are not the only issue with animal research. In recent years, greater focus on animal welfare led to the proclamation of ethical guidelines to refine, reduce and replace use of animals in research. In accordance with those “3R” principles, investigators have increased efforts to transition to more suitable *in vitro* models. Since the European court of justice banned animal experiments for cosmetic research ((EG) Nr. 1223/2009), industrial interest in alternative testing methods has also increased. There is thus a need for the development of human pruritus model systems to gain mechanistic insight into molecular itch pathways. However, these models must be physiologically accurate representations of diseases or pathways to increase translatability for clinical manifestations. A symptom-or disease-specific generation of surrogate models might be necessary to make the transition.

### Human itch studies

Regarding physiological relevance, the most obvious choice would be direct itch induction in human skin. For acute itch, intradermal histamine injection into defined body sites provides a reliable way to test histamine receptor blockers (Zhai et al., 2002). For chronic itch, the multitude of extrinsic and intrinsic factors involved in this long-lasting sensation makes a physiologically representative assessment substantially more difficult. Cowhage-induced itch is the most commonly described method to induce severe, histamine-independent itch in humans, potentially involving MRGPRX1/2 and/or PAR2 in the signaling of its pruritogenic substance mucunain (Papoiu et al., 2011; Metz et al., 2014; Reddy et al., 2018). Toxicologically-assessed, known cosmetic substances, such as polidocanol could also be tested in this model (Hawro et al., 2014a). Another possibility to induce itch in association with MRGPRD activation is the administration of β-alanine, which causes a briefer and often milder itch response compared to cowhage (Christensen et al., 2019; Klein et al., 2021). Considering the role of TRP channels in itch, trans-cinnamaldehyde, the flavor molecule of cinnamon that activates TRPA1, has been leveraged for human skin itch testing (Hojland et al., 2015).

Other studies have evoked cutaneous itch electrically and thereby gained better temporal control on pruritus generation without allergen sensitization or dependence on chemical pathways (Solinski and Rukwied, 2020). However, this mechanism without pruritogen receptor activation is perhaps best suited to observe brain activity patterns and to identify regions involved in itch processing.

Apart from electrical stimulation, the main drawback of human studies is the ethical issue of using irritants to deliberately cause itch. Sensitization and possible skin damage when applying new and putative anti-itch substances also raises ethical questions. Human itch studies are therefore limited to toxicologically approved and already well-researched substances that would require previous animal and/or *in vitro* investigations.

In general, more ways to induce itch are needed for pruritus research in different diseases and via diverse signaling pathways. For this purpose, *in vitro* models are very adaptable and could replicate essential cell–cell interactions or molecular mechanisms in pruritus otherwise inaccessible to researchers.

### Preclinical surrogate models/challenges and opportunities in *in vitro* itch research

The question arises as to what makes a good model and how complex does it need to be to fulfill its purpose? For pharmacological intervention at a specified target, monotypic cell cultures are an efficient screening tool, e.g., screening for inhibitors of a certain pruritogen receptor. However, for studies examining interactions between different cell types in disease states a more complex 2D, or 3D model is advisable.

As mentioned before, most itch research has been conducted using rodents, both *in vivo* behavioral studies and *in vitro* tests with sensory neurons, either alone or in combination with other cell types. In accordance with the 3Rs, the latter approach reduces the number of animals needed, while also increasing diversity of experiments. Despite the species differences discussed above, *in vitro* use of animal sensory neurons was for some time the only physiological way to investigate primary sensory neuron function, other than using rodent primary neuronesque cell lines, such as nerve growth factor treated PC-12 cells (Levi et al., 1988).

Ideally, human primary *ex vivo* tissue would provide the second-best option to human *in vivo* experiments for studying human chronic itch. Skin biopsies are highly accessible and include all itch relevant tissue-resident cells, as demonstrated by single cell sequencing experiments (He et al., 2020; Ahlers et al., 2021). However, the main itch-sensing cells are sensory neurons, and their cell bodies are located in the DRG next to the spinal cord, which cannot be obtained from healthy living donors, although extraction of viable cells can be achieved under certain conditions when donors are deceased or undergoing certain surgery (Valtcheva et al., 2016). Indeed, the phenotypic and functional properties of human peripheral neurons still remain poorly understood (Tavares-Ferreira et al., 2021), as extraction of human peripheral neurons remains technically and ethically challenging. Most recently, advances in the generation of sensory neurons from human induced pluripotent stem cells (hiPSCs) have enabled neuronal research beyond these limitations (Chambers et al., 2012).

However, even with a suitable cell supply, *in vitro* itch research faces challenges and limitations. Many sensory neurons equipped with pruritogen receptors also react to algogens (Klein et al., 2011; Anzelc and Burkhart, 2020). The distinction between itch and pain is likely dependent upon differential processing in the central nervous system (Forster and Handwerker, 2014) and thus any system focused purely on peripheral cell function has its restrictions. *In vitro* models completely lack the central nervous system, a crucial component for the sensation of itch (see previous section “Cellular and molecular basis of itch”), thus limiting what can be garnered from *in vitro* studies.

Most researchers therefore do not measure “itch” itself, but rather study pruritogens or receptors to uncover cellular interactions and downstream mechanisms of certain disease-associated responses. Based upon this, several *in vitro* models and methods have been developed to investigate various molecular aspects of itch, which will now be discussed.

### Methods for measuring itch-related signals

*In vitro* itch research involves numerous molecular biology, biochemical and physiological methods. In general, as the itch-transmitting organ, sensory neurons are center stage for most experiments and standard techniques in peripheral neuron research apply. The gold standard for gathering single cell drug response data is patch clamp electrophysiology, enabling measurement of changes in current flow or membrane voltage depending upon the recording configuration (Brette and Destexhe, 2012). However, most electrophysiology methods are low throughput (although automated systems, best suited to using stable cell lines, and multielectrode array measurements are changing this scenario). As a result, a commonly used, high throughput method of measuring changes in cellular excitability is Ca^2+^-imaging, which involves fluorescent based measurement of changes in intracellular [Ca^2+^]. Unlike electrophysiology, which is able to measure even very small changes in the resting membrane potential (e.g., via a Na^+^ influx), Ca^2+^-imaging is reliant on the presence of Ca^2+^-permeable ion channels, the necessary components for intracellular Ca^2+^ release from the endoplasmic reticulum, or that a depolarizing event is great enough to activate voltage-gated Ca^2+^ channels (Helmchen, 2012).

Apart from more electrophysiology and fluorescent imaging techniques that require specialized equipment, gene and protein expression analysis, as well as inflammatory cytokine or neuropeptide release are commonly used in the investigation of itch mechanisms, e.g., measuring mediator release by enzyme-linked immunosorbent assays (ELISA, e.g., substance P, TSLP, NGF or histamine).

## Histamine-dependent models

### Monotypic cell culture

As previous research demonstrated, histamine release from mast cells is the crucial mechanism for this acute form of itch. The underlying process is often simulated by histamine application to cells with endogenous or transfected histamine receptors for Ca^2+^-imaging following GPCR activation. H1 radioligand or other binding assays (Casale et al., 1985; Crane and Shih, 2004) have been adapted by commercial suppliers, who also offer simple ELISA assays or stably H1-4R transfected cell lines. See below (Histamine-independent chronic itch models) for sensory neurons that could as well be used for H1 receptor blocker tests after histamine application.

Human mast cells can be acquired for a more translational experimental model and a closer look at the release process and origin of histamine itself. In co-culture with other cells, mast cells could also act as sources of acute itch mediators (see below). Especially interesting for skin research is the isolation of primary mast cells from human skin: tissue digestion and CD117/FcεRIa+ − enrichment via flow cytometry provides viable mast cells that proliferate for ~6 weeks after isolation (Siiskonen and Scheffel, 2020).

### Co-culture

Mast cells alone are insufficient for studying itch. In co-culture with sensory neurons, researchers were able to confirm the relative importance of close-proximity communication between sensory neurons and mast cells, e.g., via the itch-associated neurotransmitter substance P (Suzuki et al., 1999, 2001). Substance P can be released from sensory neuron peripheral terminals, which causes histamine release from mast cells derived from human skin biopsies, that histamine in turn activates sensory neurons (Ebertz et al., 1987). For more information, recent reviews focusing on neuron-mast cell interactions in pruritus have been published (Gupta and Harvima, 2018; Wang et al., 2020).

## Histamine-independent chronic itch models

After the brief excursion above on acute histamine-dependent itch, the focus of this review will be on histamine-independent forms, often collectively referred to as chronic itch, a condition where H1R antagonists are inefficacious and pruritus manifests as a pathological condition.

### Monotypic cell culture

There are certain dedicated pruritus models with high complexity, but many researchers continue to use assays based on single cell types. The readout in this case is limited but does allow for conducting experiments in a highly controlled manner.

### Cell lines

Cell lines are simple to culture, typically grow rapidly and are among the simplest tools for itch research. However, the diversity of sensory neuron subtypes *in vivo* makes it largely impossible to use a single cell line that can accurately replicate the *in vivo* neuronal variety (Zeisel et al., 2018), but certain cell lines are used as sensory neuron surrogates, including: PC-12 (rat), F-11 (rat/mouse), ND7/23 (rat/mouse), ND-C (rat/mouse), 50B11 (rat), MED17.11 (mouse) and HD10.6 (human); a recent review provides details for these cell lines (Haberberger et al., 2020). Broadly speaking, each surrogate cell line features certain aspects of DRG neurons, but none are fully representative of primary cells.

Following stimulation, some ion channels and receptors provide an immediate readout (e.g., changes in [Ca^2+^] following TRP channel activation), and thus transfected cells of non-neuronal origin provide a straightforward method for investigating ion channel or receptor function and running screening assays of potential antagonists. For example, as discussed previously, TRPV3 is implicated in pruritus and its expression in HEK293T cells has provided a successful means for identifying inhibitors, such as the oral anesthetic dyclonine (found in throat sprays and lozenges), and the tropical plant-based acridone citrusinine-II (Han et al., 2021; Liu et al., 2021). Other cell lines naturally express itch related receptors, for example, HaCaT cells endogenously express PAR2 (Castex-Rizzi et al., 2014).

Overall, human cell lines may be far from accurate sensory neurons but serve a certain purpose in itch research. Transfection of pruritogen receptors allows for focused investigation but comes at the cost of not being able to observe off-target effects and/or mischaracterizing downstream signaling events. A major problem with available neuronal cell lines is that although limiting animal use, they fail to closely enough simulate DRG neuron properties (Yin et al., 2016). A particular frustration is that perhaps the only promising human DRG neuron cell line published (HD10.6), which displayed a nociceptive phenotype, seems to be the property of Celgene and is no longer available (Raymon et al., 1999; Thellman et al., 2017; Haberberger et al., 2020).

### Primary neurons

In contrast to cell lines, primary sensory neurons are equipped with the necessary pruritogen receptors, but as mentioned, access to these cells from human donors is limited. Accordingly, primary neurons are commonly isolated from rodents. Indeed, alongside what has been learned from recombinant expression systems, mouse DRG neurons have revealed a TRP-coupling mechanism for certain itch stimuli. For example, TRPV4 knockdown did not impair TRPV1-mediated Ca^2+^-responses but the other way around significantly attenuated TRPV4 function (Kim et al., 2016). This constitutes a prime example for *in vitro*-aided resolution of molecular pruritus transmission.

Since MRGPRs present an area of particular interest for chronic itch research, *in vitro* experiments with mouse DRG neurons have been utilized. For example, it was found that itch-inducing conopeptides from multiple snail venoms acted through human MRGPRX1 or mouse MRGPRC11 (Espino et al., 2018). Also, MRGPRC11 (referred to as mouse MRGPRX1) expression increases in mouse DRG neurons in a cholestasis itch model based on bile acid production. Fittingly, sensory neurons from those mice showed increased Ca^2+^-influx in response to the endogenous MRGPRX1/C11 agonist BAM8-22, which was also found to be upregulated in skin of cholestasis itch mice compared to controls (Sanjel et al., 2019).

Highly relevant for conclusions regarding neuroimmune interactions, *in vitro* tests with the Th2 cytokines TSLP and IL-31 revealed that both induced an immediate Ca^2+^-influx in mouse DRG neurons (Wilson et al., 2013b; Cevikbas et al., 2014). More recently, the same has been shown for IL-33 and IL-20, associated with dry skin itch and AD (Lu et al., 2022; Trier et al., 2022). To suppress those pruritogen responses and thereby reduce itch, JAK inhibitors proved effective by blocking downstream signaling. Results with JAK inhibitors from mouse DRG neuron studies were impressive and improved pruritus treatment options after successful translation to humans (Oetjen et al., 2017; Kim et al., 2020).

Experiments using human DRG neurons remain uncommon due to limitations of obtaining them, e.g., logistics (there is a critical window between obtaining post-mortem/surgery tissue and culturing neurons), ethical issues surrounding the use for experimental purposes, and potential legal restrictions. A pioneering study that performed *in vitro* tests with human DRG neurons incubated them with the neurotrophic factors nerve growth factor (NGF), glial-derived neurotrophic factor (GDNF) and neurotrophin-3 (NT-3). After neurotrophic factor incubation, TRPA1 responses were sensitized in a similar way to NGF-treated mouse DRG neurons (Malin et al., 2011) and to how nociceptors display increased sensitivity after injury (Anand et al., 2008). Adding to the previous work, electrophysiological analysis has found that many human DRG neurons reacted to histamine or chloroquine (Davidson et al., 2014). With relevance for chronic itch, Ca^2+^-imaging of both human and mouse DRG neurons identified that IL-31 responders react to the endogenous pruritogen endothelin-1 (Meng et al., 2018). Also in human DRG neurons, signal transmission via TRPV1 was impaired after reduced SHANK3 expression. This implied a crucial interaction between both proteins for skin sensation (Han et al., 2016). Another group investigated the function of TRPM3 in human DRG neurons and human embryonic stem cell-derived sensory neurons (hESC-SNs; Vangeel et al., 2020), the latter potentially providing a more feasible option for modeling human pruritus.

### Stem cell-based approaches

The uncovering of multiple ways to generate human sensory neurons from stem and other progenitor cells was a major leap for *in vitro* research. Peripheral neuron-like cells have been generated successfully from ESCs, induced pluripotent stem cells (iPSCs), and even through direct cell conversion.

Numerous publications describe the generation of peripheral neurons from ESCs. For example, the human neural progenitor cell line hNP1, based on ESCs, was used to derive neural crest cells (NCCs) and later electrophysiologically-active hESC-SNs (Guo et al., 2013). Electrical activity was measured after 2–4 weeks, but functional substance P expression was rarely found, even after 78 days, suggesting that these cells are not fully analogous to primary DRG neurons. In a wider structural analysis of cell properties and function over time, hESC-SNs approached more human DRG neuron-like receptor expression at d39 (Young et al., 2014). However, some sensory ion channels were overrepresented and others were missing, such as ASIC2 and Na_V_1.8, respectively. It would have been interesting to follow an extension of timepoints to evaluate when the closest DRG-like state occurred, especially regarding ion channels and receptors involved in pruritus, but as of yet, such work has not been conducted. Another hESC-based study found that spontaneous action potential firing peaked 6 weeks following differentiation, again emphasizing the necessity for prolonged maturation time (Alshawaf et al., 2018), a limiting factor for routine laboratory use.

IPSC-derived sensory neurons (iPSC-SNs) are a more widely available source for the generation of NCCs and thereafter DRG-like neurons compared to ESCs. Most iPSC-SN differentiation protocols generate P2X3-and TRPV1-positive cells after several weeks of small molecule-and growth factor-aided maturation (Chambers et al., 2012 – which still provides a good overview of the general steps required to generate iPSC-SNs). Similar to hESC-SNs, a longer differentiation time (8 weeks+) favors the functional expression of itch-related receptors, e.g., TRP channels. The general process however is time-, material-and cost-consuming, and requires improvements for increased efficiency. A recently published protocol for differentiation of iPSCs into nociceptors, proprioceptors and mechanoreceptors used Trk A/B/C antibody-immunopanning and time-displaced replating strategies (Saito-Diaz et al., 2021). Although this strategy yielded pure neuron types based on the same iPSC culture, the differentiation time of up to 10 weeks is still impractical for wholescale implementation and replacement of rodent primary sensory neurons.

Efforts are being made to further improve iPSC-based neuron culture time and receptor expression. Recent work showed that maturation time could be reduced with simultaneously higher efficiency when neurogenin-1 gene expression was switched on during development (Holzer et al., 2022). In addition to neurogenin-1, neurogenin-2 was also able to induce the conversion from NCs to a nociceptive phenotype (Hulme et al., 2020). Further emphasizing the critical aspect of time, researchers working for Pfizer described the generation of a library screening tool for higher-throughput applications using iPSC-SN. To make this possible, a shorter differentiation time was opted for at the expense of proper maturation (Stacey et al., 2018). With special focus on itch receptors, a similar, shortened approach (19 days from NCCs to SNs) was insufficient to generate a full DRG-like phenotype but still allowed for small reactions to capsaicin, AITC (TRPA1 agonist), IL-31, IL-4, and BAM8-22 (Umehara et al., 2020). With even less differentiated sensory neurons, another group have claimed to be able to test skin sensitizing substances, such as the irritant methylparaben (a possible TRPA1 agonist) by analyzing neuronal outgrowth and blebbing (Satoh et al., 2021). In addition to sensory neurons, more types of nerve cells are needed for signal transmission to the CNS, such as spinal cord interneurons, that can also be generated from iPSCs, providing a chance for downstream itch investigation and co-culture systems building the bridge to the CNS (Gupta et al., 2018).

Sensory neurons can also be generated more directly from primary cells, shortening the lengthy differentiation of stem cells. For example, epidermal NCCs can be found at the base of hair follicles and are therefore accessible neural cells from skin biopsies. Changing growth factor and small molecule exposure converted these epidermal NCCs into peptidergic sensory neurons with functional TRPV1 activation and could therefore be used as an alternative to specific primary nociceptors (Wilson et al., 2018). Neurons have also been derived from human skin precursor cells, potentially displaying the first sensory neuron phenotype obtained through direct cell conversion (Lebonvallet et al., 2012a). Unfortunately, no electrophysiological studies or functional peripheral receptor activation were conducted. Assuming these cells lacked basic features of sensory neurons, they could still be regarded as a prospect for future developments. With the same but improved method, the group achieved TRPV1 activation of those sensory neuron-like cells, although the threshold for successful activation was set very low (Bataille et al., 2020). Another readily available source of cells for direct conversion into sensory neurons is nonmobilized adult peripheral blood, which was recently established as a drug screening platform (Vojnits et al., 2019). Also, due to their spatial proximity to DRG, and supposedly plastic differentiation ability, neural crest cell-like satellite glia cells have also successfully been used for sensory neuron-conversion (Wang et al., 2021).

Although some of the above cells do not function exactly like sensory neurons generated from embryonic or pluripotent stem cells, they do benefit from being patient-specific cells and offer time-saving transformation from accessible sources.

A promising field would be the generation of hiPSC-SNs from severe skin disease patients. So far, only sensory neurons from neuropathy patients (e.g., congenital insensitivity to pain, erythromelalgia) have been verified to replicate disease characteristics mainly orchestrated via Na_V_1.7 (Meents et al., 2019; McDermott et al., 2019; Clark et al., 2021 – the latter two also provide a comprehensable methods section to generate iPSC-SNs). Additionally, iPSCs from diseased donors could help generate other cell types relevant to itch. For an accurate depiction of skin-nerve interactions in diseases characterized by pruritus, keratinocytes are essential. Contrary to cells from healthy donor biopsies, primary keratinocyte isolation from AD or psoriasis lesional skin is difficult. These cells often no longer proliferate, and it was unclear if and for how long they would maintain a lesional profile after *in vitro* culture. The hallmark Th2 cytokines IL-4 and IL-13 have been used to trigger an AD-like phenotype in human epidermal keratinocytes from healthy donors (Berdyshev et al., 2018; Dai et al., 2021). However, pluripotent stem cell-derived keratinocytes from psoriasis patients recently showed disease-specific abnormalities in differentiation and insulin resistance genes, emphasizing the role of genetic predisposing factors (Ali et al., 2020). In addition, increasing numbers of commercial suppliers are offering neural progenitor cells that can be differentiated to iPSC-SNs, thus providing further alternatives to primary animal cells. Such pre-established sensory neuron cultures can replicate some of the unique features of sensory neurons in a standardized manner and make it easier to experiment with more complex co-culture and disease models, as well as having the benefit of being human cells.

### Co-culture

As the first line of skin defense, keratinocytes take on a relevant role in signal transmission to cells underneath. Their stimulation and subsequent mediator secretion can influence nerve sprouting, immune cell behavior and itch sensation. For example, in one study, sensory neuron progenitors were differentiated for 3 weeks and subsequently cultured for 10 days with keratinocyte-conditioned medium (Guimaraes et al., 2018). Even though TRPV1 function was severely limited in the neuronal cells, the additional skin-environment-mimicking maturation step enhanced substance P release, an itch-associated neuropeptide found in late-stage iPSC differentiation (see above). However, the replating strategy used caused giant cell clusters to form, which is a major drawback for Ca^2+^-imaging or electrophysiology studies.

Other than conditioned medium, co-culture systems with cell contact provide an opportunity to study intercellular communication in a controlled environment. Indeed, electron microscopy studies show that skin-nerve communication takes place at epithelial synapse-like structures (Talagas et al., 2020c). An early skin-like co-culture model featured porcine DRG neurons and keratinocytes to mimic inflammation and pruritus by analyzing substance P secretion (Pereira et al., 2010). The same group later developed a simplified co-culture model with neuronal F-11 cells (a rat/mouse fusion cell line). Here, possibly owing to previously discussed limitations of cell lines, addition of keratinocytes had no effect on either axonal growth or Substance P release (Le Gall-Ianotto et al., 2012). In contrast, co-culture of primary rat keratinocytes with rat DRG neurons enhanced neurite extension (Ulmann et al., 2007). Further research uncovered that the effects were mediated by keratinocyte-released neurotrophins and in part the adrenal hormone dehydroepiandrosterone (Ulmann et al., 2009). Systems such as these, whereby neurons can interact with skin cells, could contribute to the growing understanding of the role of hyper−/hypoinnervation in pruritis.

### Nerve sprouting

It is controversial whether chronic itch conditions favor the sprouting or pruning of nerves in the skin. There are reports of an increased intraepidermal nerve fiber density in human AD caused by release of NGF from keratinocytes as a result of scratching. Fittingly, these publications also describe decreased levels of the nerve-retraction factor Semaphorin 3A in AD skin (Tominaga et al., 2008; Tominaga and Takamori, 2014). However, other human AD biopsy observations point to an increased length, but lower nerve density in AD (Tsutsumi et al., 2016) or a general hypoinnervation and increased pruning activity (Takahashi et al., 2019). There is potential for *in vitro* models to investigate factors affecting neuronal innervation of normal and pruritic skin.

Coculture models such as those described in the previous section can specifically measure neural sprouting using microscopy. Specifically, for other *in vitro* investigations of nerve innervation, researchers made use of specialized co-cultures that consist of sensory neurons and skin cells in compartmentalized structures. One of those devices is the Campenot chamber, microgrooves etched into plasticware permitting passage of neurites and a Teflon divider on top creates distal culture compartments (Campenot et al., 2009). Utilizing this system, porcine DRG neurons were co-cultured with human AD lesional skin cells. Compared to healthy donor cells, AD keratinocytes, but not fibroblasts, caused a stimulation of neurite outgrowth via elevated expression of NGF and GDNF (Roggenkamp et al., 2012), supporting the idea that hyperinnervation occurs in AD. Higher neurotrophic signaling and therefore nerve sensitization likely contributes to the pathological condition of chronic itch in AD.

An alternative, more basic innervation test has been established that uses Matrigel as basement membrane substitute together with an NGF gradient system in a Boyden chamber. With this setup, researchers found that matrix metalloproteinase (MMP) 2 inhibitors were effective at producing nerve retraction (Tominaga et al., 2009). In a follow-up study using collagen type I instead of Matrigel, MMP-8 expression was increased in sensory neurons and its inhibition blocked neurite extension (Tominaga et al., 2011). Both studies assumed that hyperinnervation was linked to chronic itch in AD and therefore MMP inhibitors could potentially provide effective treatment options.

## 3D and more advanced models

### Organoids and lab-on-a-chip

Microfluidic chambers (MFCs) are compartmentalized *in vitro* platforms that allow for interaction and innervation studies. MFCs are often of very small size, thus requiring relatively few cells that grow in their own defined medium, and therefore this system has proved to be a valuable addition for the transition to larger scale *in vitro* experiments.

Keratinocytes and dendritic cells have been incorporated into a closed microfluidic system with dynamic medium flow and automated trans-epithelial electrical resistance (TEER) measurements to generate an immune-competent skin model (Ramadan and Ting, 2016). This is already useful to test barrier effectiveness against irritants based on IL-6/1β expression but could also be adapted to include other cell types such as sensory neurons or Th2 cells. For more information on similar models, a recent review has been published that specifically examined co-culture systems with keratinocytes and dendritic cells (Thelu et al., 2020). Another group has created a multi-layered skin-on-a-chip format with epidermal, dermal and endothelial compartments separated by porous membranes (Wufuer et al., 2016).

While the aforementioned models could prove useful following incorporation of sensory neurons, further amendments are needed for neurite passage to enable innervation studies. In contrast, compartmentalized Xona^®^ or AXIS™ chambers harbor microchannels for neurite extension and a closed microenvironment that seemingly favors neurite growth and maturation (Nagendran et al., 2018; Kamande et al., 2019). Using the AXIS™ system with keratinocytes and DRG neurons in separate compartments, neurite retraction can be assessed and the system has potential use for screening anti-itch compounds (Kumamoto et al., 2014). Another group modified Xona^®^ chambers to gain access to the neuronal cell bodies for electrophysiology and Ca^2+^-imaging. This added the ability to apply drugs in the compartment with rat neonatal keratinocytes and innervating axons, while measuring the effect in the opposite somal chamber (Tsantoulas et al., 2013). A more recent study used hiPSC-derived sensory neurons together with human keratinocytes in Xona^®^ devices to study cutaneous skin afferent communication, and concluded that neurites were attracted by keratinocytes in co-culture (Belamadni et al., 2022).

Advancing from lab-on-a-chip models, organoids are considered highly promising. Researchers have used mouse pluripotent stem cells in an organoid system and observed self-assembly and differentiation of skin layers. After several weeks, sebaceous glands and hair follicle formation occurred, as well as neural crest-like cells, showing the potential for eventual sensory neuron manipulation to create a whole skin itch model (Lee et al., 2018). With even more physiological relevance to the human system, the same researchers later used hiPSCs to successfully create similar self-assembled and fully stratified skin organoids including hair follicle formation (Lee J. et al., 2020, 2022; Ramovs et al., 2022). In another highly complementary organoid system, hESC-SNs were introduced to endothelial cells and proved essential for vascular formation (Kannan et al., 2021). This again underlined the relevance of 3D tissue modeling for skin (disease) research.

### 3D skin models

To achieve skin-like stratification and epidermis formation, keratinocytes can be cultured in 3D at an air-liquid interface. This reconstructed human epidermis was tested in the context of sensitive skin with chemically-induced itch. Lactic acid treatment decreased TEER and barrier protein gene expression (e.g., filaggrin), while neurotrophin genes such as brain-derived neurotrophic factor (BDNF) and artemin (ARTN) were upregulated, indicative of increased skin irritation (Hasan et al., 2019). Another comparable test with reconstructed epidermis and inflammatory cytokine release assay concluded that few-layer graphene is non-irritant, but this system has not yet been used for the systematic study of itch (Fusco et al., 2020). However, given the single cell type and no interaction with immune or nerve cells, this model should not be considered an accurate depiction of skin, which will limit conclusions that can be made. With the addition of fibroblasts, a system can be referred to as “full-thickness skin equivalent” and could then prove more valuable for investigation of pruritic skin diseases. This could also be done entirely from patient-derived iPSCs (Itoh et al., 2013).

As mentioned before, AD keratinocytes are difficult to extract from lesional skin biopsies and exhibit a low proliferation rate. However, full-thickness explant culture models have shown that cells maintained their disease profile *in vitro*, including barrier defects (Van Drongelen et al., 2015). Others have suggested that AD fibroblasts are even more essential for AD modeling, various 3D skin models with healthy and AD skin cells showcasing the reduced secretion of leukemia inhibitory factor (LIF) by atopic fibroblasts (Berroth et al., 2013). Both these studies provide a basis for physiologically representative investigations without the need for artificial AD-like cytokine treatment. Examples of such cytokine treatments are FT-HSE incubation with methyl-β-cyclodextrin and IL-4, as well as other models using a combination of IL-4, −13, −31 and TNF-α (Danso et al., 2014; Do Nascimento Pedrosa et al., 2017; Sriram et al., 2019). A unique way to rebuild itch-related skin diseases in 3D includes the incorporation of Th1 and Th17-polarized T cells, which induced psoriasiform inflammation and keratinocyte differentiation (van den Bogaard et al., 2014). For a recent review on models for understanding mostly the inflammatory side of AD and psoriasis, see (Sarama et al., 2022).

Researchers have also developed bioprinted, multi-well 3D skin models comprising keratinocytes, fibroblasts, pericytes and endothelial cells to allow for skin vascularization. Strikingly, JAK inhibitor tests in these models reversed an IL-4 induced AD phenotype and even increased epithelial resistance (Liu et al., 2020). For further advanced treatment options in AD, 3D miniature organotypic skin models were treated with the small molecule osthole, which inhibited TLR2 signaling involved in *S. aureus*-induced itch (Kordulewska et al., 2021).

Adding to the already introduced *in vitro* models, a recent review described 2D to 3D models with keratinocytes and dendritic cells for the assessment of skin sensitization and thereby itch in allergic contact dermatitis (Thelu et al., 2020). For example, as a specialized subset of dendritic cells and due to their residence in the epidermis, the incorporation of Langerhans cells into a full thickness skin equivalent was especially useful to observe skin sensitization events (Bock et al., 2018).

Starting in 2003, researchers began to explore the potential of innervated 3D skin models with mouse DRG neurons for neurite growth studies and found that keratinocytes are crucial for neurite survival (Gingras et al., 2003). As described in the co-culture section above, keratinocyte-nerve communication is an essential transmission pathway, and it indeed works both ways. Epidermal growth gets stimulated by CGRP and Substance P release from porcine DRG neurons (Roggenkamp et al., 2013).

Another group have used the HaCaT keratinocyte cell line for epidermis formation on a self-assembled fibroblast dermis. This sophisticated approach utilized computational fluid dynamics for optimal 6–10 weeklong culture conditions. Additionally, collagen assembly was observed using second harmonic generation imaging. In this system, epidermis-directed rat DRG neuron innervation could be illustrated with two-photon fluorescent imaging and TRPV1 functionality was shown after 8 days of innervation (Martorina et al., 2017).

**Figure 2:**
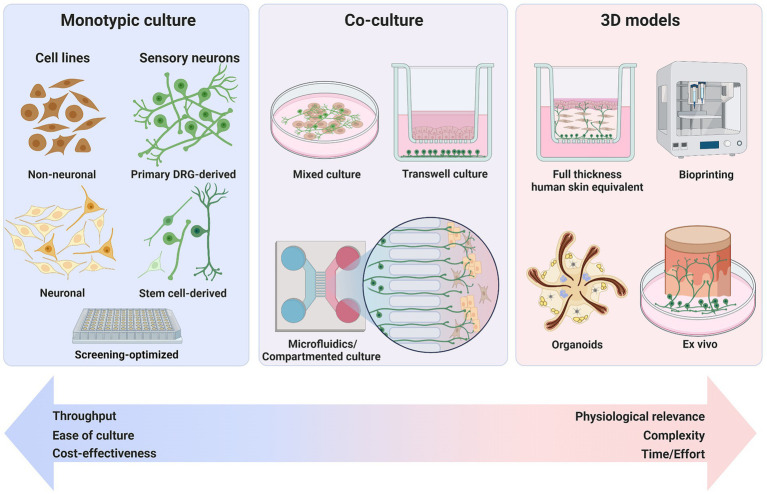
Types of *in vitro* models for investigating itch. From simplistic monotypic 2D cultures to more elaborate 3D setups, surrogate models have been developed for the depiction of mechanisms that characterize itch. Physiological relevance for translation to the human system increases with complexity of the models but comes at the cost of lower throughput, higher cost, and greater effort. Created with BioRender.com.

The first innervated human 3D skin model with iPSC-SNs included primary fibroblasts, keratinocytes and also endothelial cells in a collagen sponge scaffold. It was observed that mouse or iPSC-derived Schwann cells underneath the epidermis were necessary for epidermis-directed neurite growth (Muller et al., 2018). With such a multitude of cell types in 3D, the measured substance P expression presented a physiologically accurate readout in response to topical compound applications. Compared with other iPSC-SN protocols, the short neuronal maturation time could have affected the neuronal phenotype, but the chosen time course likely reflects survival of the whole model. Those last systems embodied prime examples for increased complexity and effort in skin modeling to be used for itch research. Nevertheless, it is important to note that the addition of more cell types also limits scalability, adds cost, decreases throughput and might make reproducibility across research laboratories more difficult (Figure 2).

### *Ex vivo* models

In comparison with human nerve tissue, skin biopsies are easily accessible and can be utilized for *ex vivo* models. For example, researchers presented a pharmacological tool comprised of skin explants, monocyte-derived dendritic cells (MoDCs) and autologous T cells for drug hypersensitivity reactions. Co-culture of MoDCs pre-exposed to drugs of differing mechanisms of action (e.g., carbamazepine, ofloxacin, lapatinib, all of which are low molecular weight drugs for which the assay was developed) primed the T cells that migrated into the skin biopsy afterwards (Ahmed et al., 2019). T cell proliferation scores and a higher IFNγ production in the explant were indicative of a cutaneous immune response that could have triggered itch for multiple of the tested drugs. However, the addition of sensory neurons and their activity profile would have allowed for an even better conclusion on drug hypersensitivity.

To address the missing neurons in skin biopsies, some researchers have added PC-12 cells to skin explants. This nerve-skin setup can be used to analyze the differential effects of skin biopsies from healthy or disease patients (Lebonvallet et al., 2013). The same group also performed re-innervation with rat DRG neurons, which decreased apoptosis of keratinocytes in skin explants, thus suggesting an essential role of neuronal innervation for epidermal integrity and survival (Lebonvallet et al., 2012b). This once more emphasizes the importance of bidirectional keratinocyte-neuron signaling. Using the same explant system, rat DRG neurons showed an increase in electrical activity in patch-clamp recordings when capsaicin was applied directly onto the epidermis (Lebonvallet et al., 2014). Interestingly, the neurons did not respond to capsaicin when applied to the surrounding medium. Therefore, skin cells were thought to have transmitted molecular signals in reaction to capsaicin even though it is debatable as to what extent keratinocytes express capsaicin-sensitive TRPV1 (Pecze et al., 2008). In the most recent iteration of this skin-nerve model, topical administration of TRPA1 and PAR2 agonists (polygodial and SLIGKV, respectively) also increased electrical activity. However, gene expression analysis and TSLP/CGRP release assays after agonist application revealed mixed results (Lebonvallet et al., 2021). Nevertheless, this model appears to be especially useful to look at neuronal stimulation by potentially pruritus-inducing compounds in a physiological *in vitro* environment.

## Discussion and outlook

Recent advances in itch research have generated multiple opportunities to study itch mechanisms *in vitro* and have also resulted in many new insights into this complex sensation. Sensory neurons are the primary itch-sensing cells and interact with virtually all skin and resident immune cells to generate itch signals. Intercellular communication is characterized by constant exchange of cytokines, neurotransmitters, and neurotrophic factors, which is likely altered in pruritic skin diseases such as AD. Therefore, understanding intercellular processes in health and disease is critical to developing new therapies, *in vitro* methods offering numerous advantages over *in vivo* studies for such detailed analysis.

Comprising sensory neurons, skin and immune cells, 2D and 3D co-culture models, skin reconstructions and even explant systems have been used to investigate itch. The more human-cell-based and complex a model that is developed, the more accurate its depiction of the primary system in humans for whom treatment is sought. However, a major limitation of cellular systems is the missing connection to the CNS. However, blocking pruriception at its source might be the most efficient way of preventing itch. That is one reason why researchers have focused largely on identifying and analyzing specific pruritogen receptors and the unraveling of intra-and intercellular signaling pathways in cellular systems.

For a physiologically accurate depiction of pruritogen signaling pathways, primary human DRG neurons have recently gained greater use. However, logistical problems provide a major hurdle preventing routine use of human DRG neurons, but they provide an excellent model for final mechanism or compound validation. The newly emerging field of iPSC-SN biology has come a long way, with ever more rapid and effective maturation/differentiation protocols being developed. However, there are still several limitations of iPSC-SNs, perhaps most significantly their failure to accurately recapitulate the diversity of human DRG neuron subtypes, as well as the arduous and costly maturation time still lasting weeks.

It is to be expected that *in vitro* models for studying itch will develop alongside new insights into pruritic diseases. For tailoring specific models to test treatment options, suitable targets need to be identified first. It is likely that different types of chronic itch will require different directed approaches, as pruritic conditions are highly heterogenous. At this moment, as it is still unclear for many pruritic conditions what exactly causes the malfunctioning skin sensation, omics studies could help to reveal relevant therapeutic targets. For example, new findings in human healthy vs. disease donor biopsies via single cell sequencing are likely to uncover contributions of different cells to lesional and itchy skin states (He et al., 2020, 2021).

Adding to more disease-specific challenges in itch research, personal medicine applications will grow in importance along with patient-derived cells for disease modeling and identification. Even the differences between iPSC-SN derived from different healthy individuals cultured with the same protocol emphasizes that indeed one model system might not fit all (Schwartzentruber et al., 2018). This obviously affects treatment options as well. Another challenge for non-explant *in vitro* models is the variety of cell subtypes found in human skin. Keratinocytes are often treated with 3D differentiation medium and an air-liquid interface culture is performed for epidermal stratification. However, fibroblasts are so heterogenous throughout the dermis that even primary isolated and *in vitro* expanded fibroblasts do not accurately replicate the *in vivo* situation in organotypic skin models (Sriram et al., 2015).

Despite the challenges, itch research has come a long way, including the numerous *in vitro* experiments and methods that have enhanced our understanding and covered diverse aspects of this peculiar skin sensation. Most *in vitro* experiments provide the possibility to study disease mechanisms without requiring access to patients or primary tissue. They also facilitate experimental scalability, enable low-cost setups, and permit direct observation and measurement of intercellular communication. Therefore, even considering their limitations, *in vitro* models are valuable tools to unravel the various mechanisms of itch in preclinical research.

Looking to the future, we can expect to see more entirely human *in vitro* models, which inherently have greater physiological relevance and clinical translatability. As mentioned above, human DRG neurons and skin are different to those of rodents regarding structure, cell phenotypes and protein expression. However, rodent pruritus research has also proven valuable for development of itch treatment options in companion animals. For example, dogs and cats also develop AD or similar pruritic diseases, which can pose a heavy burden on both pets and owners (Gedon and Mueller, 2018). For that, the JAK inhibitor oclacitinib was FDA-approved in 2013 (Gonzales et al., 2014), nearly a decade before JAK inhibitors gained approval for AD treatment in humans.

Currently, it is not possible to completely substitute *in vivo* work with only *in vitro* models. Firstly, because itch is a complex process involving numerous organ systems including interactions between the peripheral and central nervous systems. Secondly, because *in vivo* experiments will always be required to obtain data regarding, for example, the pharmacokinetic properties of novel therapeutics. However, *in vitro* approaches are not only able to offer significant insights, but also help to reduce the number of animals used in experiments.

Alongside *in vitro* cellular systems, computational and *in silico* advances, together with the development of artificial intelligence and deep learning approaches could prove extremely useful for pruritus research (Rifaioglu et al., 2019). With assistance from computer-aided research, the general need for initial testing of new antipruritic substances using traditional “wet lab” techniques could significantly decrease (Chandra et al., 2020; Clayton et al., 2021; Sliz et al., 2022).

From simple monotypic cell cultures to complex re-innervated skin, *in vitro* itch models have steadily evolved to fit the need for preclinical *in vitro* investigations. With rapid advances in hiPSC-SN biology, the growing experimental use of primary human DRG neurons, and development of *in silico* approaches, itch research is expected to be ever more characterized by the use of sophisticated and elaborate *in vitro* methodology. This will help to refine and reduce the number of *in vivo* studies, and aid to improve therapeutic solutions for people suffering from chronic itch.

## Author contributions

HM wrote the initial draft of the manuscript that was edited by JS and ES. All authors contributed to the article and approved the submitted version.

## Conflict of interest

JS is an employee of Beiersdorf AG. HM was formerly employed by Beiersdorf AG, and ES has received funding from Beiersdorf AG in the past.

The authors declare that this affiliation did not influence the current work and this study was conducted in the absence of any commercial or financial relationships that could be construed as a potential conflict of interest.

## Publisher’s note

All claims expressed in this article are solely those of the authors and do not necessarily represent those of their affiliated organizations, or those of the publisher, the editors and the reviewers. Any product that may be evaluated in this article, or claim that may be made by its manufacturer, is not guaranteed or endorsed by the publisher.
